# MPT64 patch test for the diagnosis of active pulmonary tuberculosis: a randomised controlled trial in Peru

**DOI:** 10.5588/ijtld.17.0716

**Published:** 2018-06-01

**Authors:** V. Pope, K. A. Sacksteder, J. Coronel Hererra, R. H. Gilman, S. Vargas-Prada, S. Lopez Romero, J. Yafac, E. Sanchez Rios, D. A. J. Moore

**Affiliations:** * TB Centre, London School of Hygiene & Tropical Medicine, London, UK; † Sequella, Inc, Rockville, Maryland, USA; ‡ Universidad Peruana Cayetano Heredia, Lima, Peru; § Johns Hopkins Bloomberg School of Public Health, Baltimore, Maryland, USA; ¶ Centre for Research in Occupational Health, Universitat Pompeu Fabra, Barcelona, Spain; # Unidad Central de Contingencias Comunes (U3C), Mutua Asepeyo, Barcelona, Spain; ** Hospital Huaycan, Lima Este, Peru

**Keywords:** TB, diagnostic, MPT64, patch test

## Abstract

**SETTING:**

There remains a lack of effective and inexpensive diagnostic tools for active tuberculosis (TB) disease. Testing immune responses to proteins secreted by Mycobacterium tuberculosis, such as MPT64, may be a diagnostic option.

**OBJECTIVE:**

To evaluate the sensitivity and specificity of a patch test using MPT64 for the diagnosis of active TB disease.

**DESIGN:**

This randomised, double-blind, placebo-controlled, prospective study in Lima, Peru, involved 55 healthy controls and 457 symptomatic individuals referred for routine TB testing by the National TB Control Programme. All subjects underwent a comprehensive diagnostic workup, and received an active patch on one arm and a placebo patch on the opposite arm, which were read after 4 days.

**RESULTS:**

Eighty-one (18%) of the symptomatic participants were classified as having definite TB, while an additional 98 (21%) had probable TB. The patch tests performed the same in both groups, with a sensitivity of 27% and specificity of 74%. The area under the receiver operating characteristic curve was 0.495 (95%CI 0.425–0.565).

**CONCLUSIONS:**

Contrary to existing literature, the MPT64 patch was not sensitive and specific to detect active TB. Given the potential of the test, understanding possible differences in the protein source or underlying genetic factors should be explored further.

## Abstract (FR)

**CONTEXTE:**

Il manque toujours des tests de diagnostic fiables et peu coûteux de la tuberculose (TB) active. Tester les réponses immunitaires aux protéines sécrétées par Mycobacterium tuberculosis, comme MPT64, pourrait être une option de diagnostic.

**OBJECTIF:**

Evaluer la sensibilité et la spécificité d'un patch test basé sur MPT64 pour le diagnostic de la TB active.

**SCHÉMA:**

Cette étude prospective randomisée, en double aveugle, contre placebo, à Lima, Pérou, a impliqué 55 témoins en bonne santé et 457 individus symptomatiques référés pour un test TB de routine par le Programme National de lutte contre la TB. Tous les sujets ont eu un bilan diagnostic complet et ont reçu un patch actif sur un bras et un patch placebo sur le bras opposé ; la lecture a été faite après 4 jours.

**RÉSULTATS:**

Des participants symptomatiques, 81 (18%) ont été classés comme ayant une TB certaine, tandis que 98 autres (21%) avaient une TB probable. Les patch tests ont eu la même performance dans les deux groupes, avec une sensibilité de 27% et une spécificité de 74%. La zone sous la courbe de la fonction d'efficacité du récepteur a été de 0,495 (IC95% 0,425–0,565).

**CONCLUSION:**

Le patch MPT64 n'a été ni sensible ni spécifique dans la détection de la TB active, en contraste avec la littérature existante. Etant donné le potentiel de ce test, comprendre les différences potentielles de la source de protéines ou les facteurs génétiques sousjacents devrait être davantage exploré.

## Abstract (ES)

**CONTEXTO:**

En la actualidad no se dispone de métodos diagnósticos efectivos y de bajo coste para la enfermedad activa por tuberculosis (TB). Analizar la respuesta inmune a proteínas secretadas por Mycobacterium tuberculosis, como la MPT64, podría ser una opción diagnóstica.

**OBJETIVO:**

Evaluar la sensibilidad y especificidad de una prueba de parche que utiliza MPT64 para el diagnóstico de la enfermedad activa por TB.

**DISEÑO:**

Este estudio prospectivo, aleatorizado, doble ciego, controlado con placebo en Lima, Perú, incluyó a 55 controles sanos y 457 individuos sintomáticos remitidos al Programa Nacional de control de la TB para pruebas rutinarias de detección de TB. Todos los sujetos fueron sometidos a un examen diagnóstico exhaustivo y recibieron un parche activo en un brazo y un parche de placebo en el brazo opuesto, los cuales fueron leídos después de 4 días de colocados.

**RESULTADOS:**

De los participantes sintomáticos, 81 (18%) fueron diagnosticados como TB definitiva, mientras que 98 (21%) como TB probable. Los resultados de la prueba del parche fueron similares en ambos grupos, obteniendo una sensibilidad del 27% y una especificidad del 74%. El área bajo la curva característica operativa del receptor fue 0,495 (IC95% 0,425–0,565).

**CONCLUSIONES:**

En contraste con la literatura existente, el parche MPT64 no fue sensible ni específico para detectar TB activa. Dado el potencial de la prueba, se debería explorar con más detalle las potenciales diferencias en la fuente de la proteína y los factores genéticos subyacentes.

IN 2016, TUBERCULOSIS (TB) incidence was estimated at 10.4 million cases worldwide, and resulted in 1.3 million deaths.^1^ TB remains an important global health problem, but effective control is hampered by the lack of an effective, inexpensive and readily scalable diagnostic tool to distinguish between active disease, latent infection and past exposure or vaccination. There is a need for improvement in the diagnosis of active disease, as delay in diagnosis both worsens the prognosis for the individual and permits ongoing transmission in the community.^2–4^

Tools currently employed to diagnose tuberculous infection or TB disease include the tuberculin skin test (TST) using a purified protein derivative (PPD), sputum smear microscopy and culture, interferon-gamma release assays (IGRAs) and molecular assays such as Xpert^®^ MTB/RIF (Cepheid, Sunnyvale, CA, USA) polymerase chain reaction amplification. However, there are constraints that limit the usefulness of each tool for diagnosing active disease. TST and IGRAs cannot reliably distinguish between infection and disease,^5–8^ while the low sensitivity of sputum smear microscopy means it can detect only 50% of active pulmonary cases and its limited specificity means that Mycobacterium tuberculosis cannot be distinguished from non-tuberculous mycobacteria (NTM).^9^ Sputum culture overcomes this limitation, but it takes at least 1 week to obtain a result and requires a biosafety level 2 laboratory.^9^,^10^,^11^ Although Xpert obviates the need for a laboratory and can provide rapid detection and rifampicin susceptibility results, the equipment and cartridge costs, the fragility of the platform and the continued need for laboratory infrastructure for downstream drug susceptibility testing impedes the sustainability and capacity for peripheral distribution in endemic regions.^9^,^12–14^

The MPT64 patch test was developed by Nakamura et al. to address the above limitations.^15^,^16^ The test consists of a standard gauze patch with a solution containing purified MPT64 protein. It is placed on the volar surface on either forearm, and the skin reaction is read after 4 days. Positive reactions consist of a dermal reaction to the MPT64 protein, usually erythema, induration or small red vesicles at the patch site. Small Phase I and Phase II studies of a patch test by Nakamura et al. demonstrated excellent sensitivity (>85%) and specificity (100%), while distinguishing active disease from exposure or vaccination in asymptomatic humans.^15^,^16^ These trials used MPB64 extracted from liquid culture filtrate produced as a byproduct of growing bacille Calmette-Guérin (BCG) Tokyo vaccine culture. Each patch required approximately 1.5 l of BCG, thereby limiting the potential for application to large-scale trials or future deployment.^5^ However, MPT64, the protein secreted by M. tuberculosis (rather than M. bovis), is identical to MPB64 at the amino-acid level, and can be produced at scale by purifying it from recombinant Escherichia coli. At the time of the above clinical trial, it was demonstrated that this recombinant (r) MPT64 performed identically to MPB64 in a guinea pig model, and so rMPT64, produced by Sequella Inc. (Rockville, MD, USA) under Good Manufacturing Practices, was used for the trial instead of MPB64. Encouraged by these early findings, our study was undertaken to assess the sensitivity and specificity of the MPT64 patch test in the diagnosis of active TB disease in a Phase III clinical trial.

## METHODS

### Study setting and design

This was a randomised, double-blind, placebo-controlled, prospective study. Both healthy and symptomatic patients were recruited from health care establishments in Lima, Peru, from February to December 2005. Group 1 consisted of 75 healthy controls with no signs or symptoms of TB. Group 2 comprised 450 symptomatic individuals referred for routine testing for TB by the National TB Control Programme. All subjects underwent a comprehensive diagnostic workup and received an active patch on one arm and a placebo patch on the opposite arm, as detailed below.

### Study participants

Consecutive patients aged 18–65 years who provided voluntary informed consent were included in the study. Symptomatic patients either visited the clinic directly or were referred from a feeder clinic for investigation of respiratory symptoms, including TB testing. Healthy controls were recruited if they reported no human immunodeficiency virus (HIV) infection or active TB within the previous 2 years, no regular contact with a person with active TB and were sputum smear negative for acid-fast bacilli.

### Study procedures

Demographic and clinical details were collected and all subjects underwent a complete physical examination and chest X-ray. HIV testing was performed with CD4 count for subjects known to be or newly diagnosed with HIV infection. Two sputum samples were collected and submitted for auramine fluorescence smear microscopy and liquid culture using the microscopic observation drug susceptibility assay.^17^ Patches were applied, one to each forearm, and patients returned 96 h later to have their patches removed and read, and to undergo TST. Subjects returned 48 h later for TST reading and a further reading on day 6 of the two patch test sites. Follow-up visits to repeat all procedures were originally planned for months 3, 9, 12, 15 and 18.

### The MPT64 patch

The MPT64 patches consisted of two component parts: a Torii patch (Torii and Co, Tokyo, Japan) and MPT64 antigen in phosphate-buffered saline (PBS) solution. The previous MPB64 patch test studies had used PBS and 0.005% Tween-80 as a buffer mixture; however, as in the pilot studies in Peru mild skin reactions to this buffer mixture alone were frequent, Tween-80 was not used here. Placebo patches were included so that reading would be blinded and, if a non-specific reaction to the buffer and patch occurred, a differential response could be recorded.

Placebo patches contained only the Torii patch and buffer without MPT64. The patches were an adhesive plaster with round, disc-shaped gauze 17 mm in diameter and 1 mm thick at the centre. Immediately before patch application, 100 μl of either MPT64 (total dose 150 μg) or placebo solution was applied to the gauze of the Torii patch.

Randomisation was performed by Sequella Inc., which filled 10 tubes for each patient with either MPT64 or control solution and randomly assigned a label corresponding to an arm and subject number (e.g., 1A/1B, 2A/2B). Solution A was applied to the right arm patch, and Solution B to the left arm patch. Research nurses preparing and applying the patches were blinded to which solution contained the active antigen. At the time of patch reading, staff were blinded to TB status because of the time required to conduct confirmatory culture tests. Patch results were photographed and recorded as positive or negative before knowing the TB status.

### Patch reading

After removal of the patch at 4 days (if still adherent), the patch sites were evaluated for erythema, vesiculation, pruritus and induration subjectively by the study nurse or physician, and were graded by the study staff as part of an exploratory objective scale. Participating subjects were also asked to evaluate the responses, with particular attention given to any differential reaction.

### TB definition and assignment

Participants were assigned TB status from one of the following: definite TB, probable TB, and not TB. Definite TB was defined as a patient with any positive microbiological test among two sputum microscopy tests and two sputum cultures. Probable cases were defined as individuals with chest X-ray findings consistent with TB who started on TB medication within 3 months of the initial visit, or cases with a diagnosis of TB made elsewhere within 3 months of the initial visit, but without microbiological confirmation.

### Data analyses

**Figure A.1:**
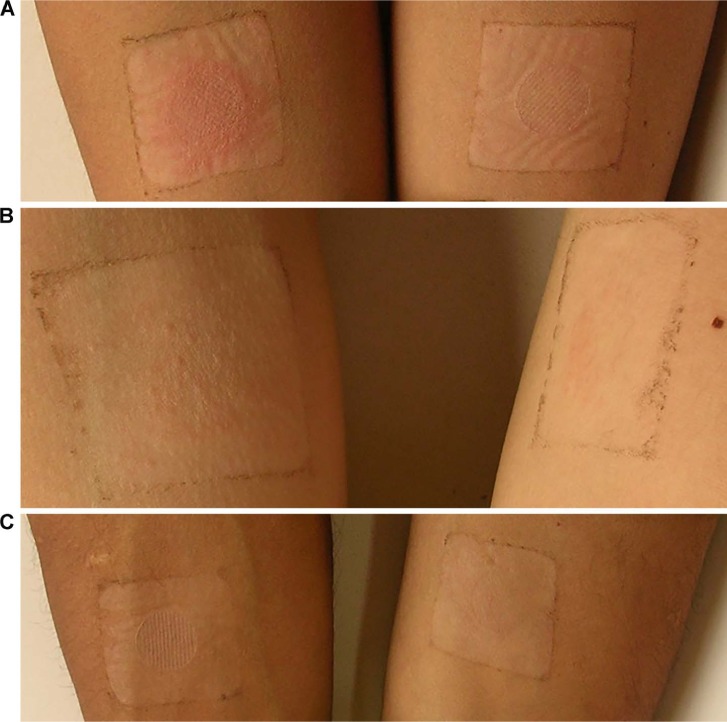
Examples of A) a positive, B) an indeterminate and C) a negative patch test.

Analyses were conducted using definite active TB cases only, and then using both definite and probable cases. The main outcome measure of interest was performance of the patch test in distinguishing active TB from not TB, as measured by sensitivity and specificity, according to assessment by field staff. Patch test readings were designated ‘inconclusive’ if there were cutaneous reactions on both forearms without a qualitatively discernible difference (in other words, the placebo patch provoked a reaction that was qualitatively indistinguishable from the MPT64 patch) or if there was a markedly greater reaction on the placebo site. These inconclusive patches were not included in the analysis. The remaining patches were designated ‘positive’ if only the active patch site demonstrated a reaction, and ‘negative’ if neither patch evoked any reaction (Appendix Figure A.1^*^). Analysis of participant self-evaluations was included to explore the feasibility of self-testing. An exploratory dermal reactivity scale was developed to establish an objective cut-off value for positive tests.

Sample size was calculated using NQuery Advisor 5.0 (Statistical Solutions Ltd., Cork, Ireland) using the exact test for a single proportion. All calculations were done using α = 0.05 and assuming a two-sided test. A target sample size of 90 definite TB cases was chosen to provide 83% power for a lower confidence limit for a sensitivity of >85%, assuming that true sensitivity was ⩾95%. Using local data, it was assumed that only 20% of symptomatic individuals would have bacteriologically confirmed TB; the actual sample required was therefore 450 patients undergoing investigation for TB. A sample size of 75 controls was chosen to provide 81% power for a lower confidence limit for a specificity of >90%, assuming that true specificity was ⩾98%.

Statistical analysis was conducted using Stata for Mac 13 (StataCorp, College Station, TX, USA). Basic descriptive statistics were used to summarise continuous and categorical data. Two-by-two tests of case status and patch status were performed, followed by receiver operating characteristic (ROC) curves of the objective patch results to determine a cut-off value. ROC regression was used to adjust for age, sex and HIV status. Reporting was conducted in accordance with Standards for Reporting of Diagnostic Accuracy (STARD) guidelines.

### Ethical review

The study protocol was approved by the institutional review boards of Hammersmith and Queen Charlotte's & Chelsea Research Ethics Committee in London, UK, and the Universidad Peruana Cayetano Heredia in Lima. Institutional approval was granted by the Direccion de Servicios de Salud (DISA) Lima Sur and ′ DISA Lima Este of the Peruvian Ministry of Health.

## RESULTS

### Baseline characteristics of study participants

Participant enrolment resulted in the recruitment of 457 subjects undergoing investigation for TB and 55 healthy, asymptomatic individuals. The mean age of the symptomatic participants was 32.3 years, with three quarters of participants aged ⩽40 years and a slight female preponderance (Table 1). HIV prevalence was low, at 3%; of these, 38% had a CD4 count <200 cells/mm^3^. Although many patients also had concurrent comorbidities, only two had a skin condition that might have affected the patch response. Loss to follow-up after 4 days was small, with 497 of 512 (97%) subjects returning for review.

**Table 1:**
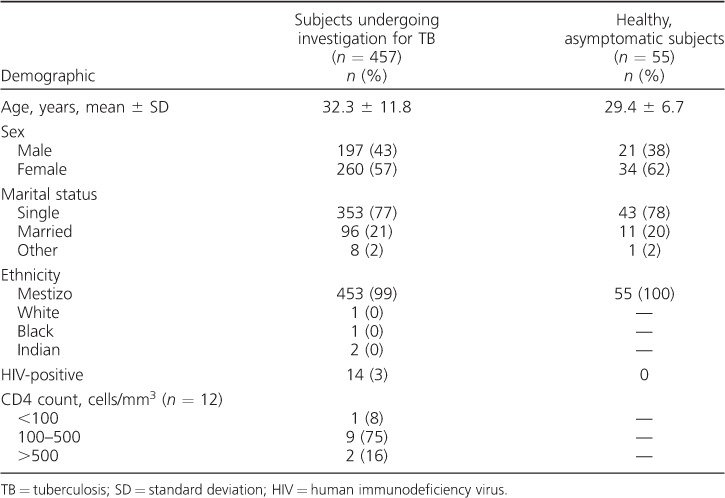
Demographic characteristics of the study population

Of 457 participants, 81 (18%) undergoing investigation for TB were classified as having definite TB (Figure, A); 179 (39%) had definite or probable TB. Sputum smear microscopy was positive in 51 participants (11%). All healthy, asymptomatic subjects were classified as not having TB (Figure, B). Criteria used for classification are shown in Table 2.

**Figure:**
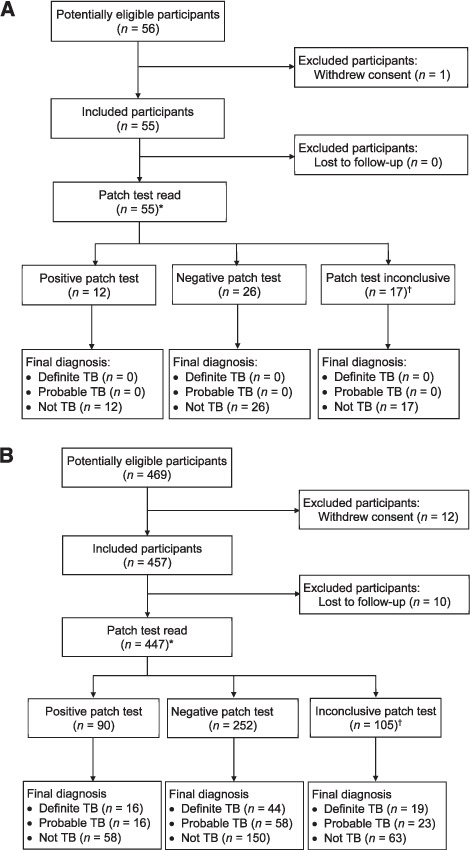
A) STARD flowchart for subjects undergoing investigation for TB (patch test reading by a trained research nurse). ^*^ 70 participants were missing one or more patches at the time of follow-up. These were included in the final analysis as the test could be read from the dermal reaction before the patch was lost. A separate analysis (not shown) found no difference between including only fully attached patches and including everyone who returned after 4 days. ^†^ Placebo patch reaction (n =97), reaction in both patches (n = 8). B) STARD flowchart for asymptomatic healthy individuals (patch test reading by a trained research nurse). ^*^ 7 participants were missing one or more patches at the time of follow-up. These were included in the final analysis as the test could still be read from the dermal reaction before the patch was lost. A separate analysis (not shown) showed no difference between including only fully attached patches and including everyone who returned after 4 days. ^†^ Placebo patch reaction (n= 10), reaction in both patches (n =7). TB =tuberculosis; STARD = Standards for Reporting of Diagnostic Accuracy.

**Table 2:**
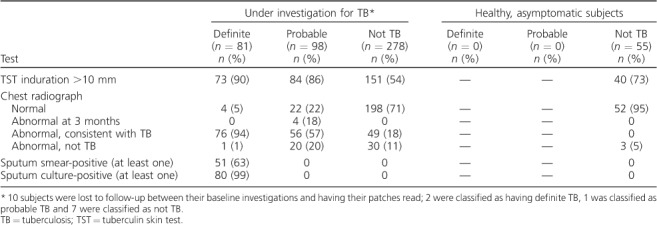
Reference criteria for TB category assignment

### Performance characteristics

Applying the optimal conditions for performance (outcome only including definite TB, trained staff evaluation), the sensitivity and specificity in distinguishing active TB from no TB among individuals being investigated for TB were respectively 26.7% and 74.1% (Table 3). The use of different case definitions or evaluators had no important effect upon these parameters. Specificity among healthy asymptomatic participants without TB was 68.4% when evaluated by trained staff and 60.5% when subjects self-evaluated.

**Table 3:**
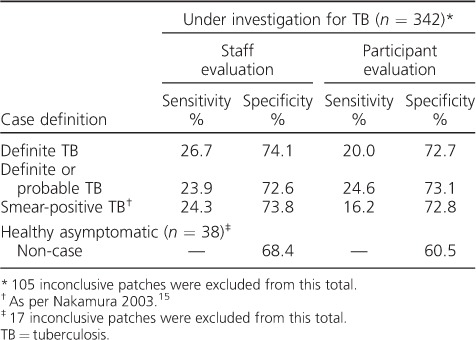
Sensitivity and specificity analysis (evaluable participants only)

### Receiver operating characteristics of dermal reactivity scale

**Figure A.2:**
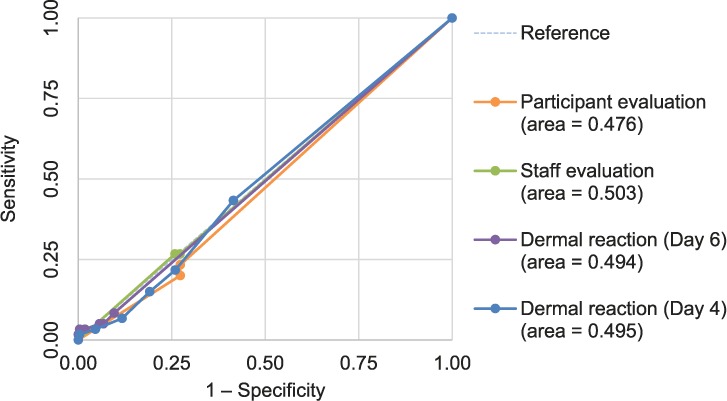
Comparison of receiver operating characteristic curves for various evaluation methods (participants under investigation for tuberculosis only, n = 342).^* *^ 105 inconclusive patches were excluded from this total.

There was a clear trend of decreasing frequency at each level of the objective dermal reactivity scale (Table 4), suggesting that the ordinal scale reflected increasing reactivity. However, ROC curve analysis did not reveal an obvious cut-off point for a positive test result. The dermal scale, as reported after 4 days, provided an area under the curve (AUC) value of 0.495 (95% confidence interval [CI] 0.425–0.565) (Appendix Figure A.2). Other evaluation methods, such as staff or participant evaluation, did not perform better. Restricting analyses to include only those subjects whose patches remained fully attached or including probable TB cases did not have a significant impact. Finally, adjustment for age, sex and HIV status reduced the AUC to 0.421.

**Table 4:**
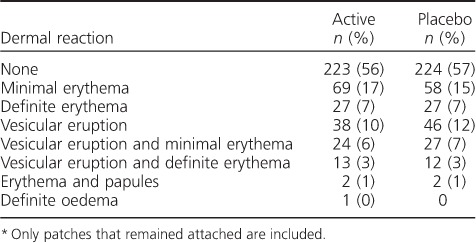
Dermal reactivity scale and results (subjects under investigation for tuberculosis only, n = 397) ^*^

### Positive patch tests

Of the 209 participants with one patch reaction markedly stronger than the other, regardless of TB status, the MPT64 patch was correctly identified as the active patch in 102 (49%) subjects. In the remaining 107 (51%), the test was erroneously deemed as being positive on the basis of a stronger reaction to the placebo patch.

### Adverse events

Detailed data on all adverse events were collected. Adverse events were recorded for 36% (184/512) of participants. Each subject with an adverse reaction had an average of 1.6 events, 11% of which were characterised as serious: 16 subjects required hospitalisation, 3 died and 1 suffered significant or persistent disability. However, none of these events were considered related to the patch test. Of the 350 total events, only nine (3%) were deemed possibly or probably related to the patch test. The most common patch reactions were itching (*n* =3, 33%) and allergic dermatitis (*n* = 2, 22%). Pain, discomfort, burning sensation, infection at the patch site and pigmentation disorder each occurred once. Finally, two subjects reported a transient, sharp pain at the placebo site only.

## DISCUSSION

The key finding of this double-blind, placebo-controlled trial was that the MPT64 patch test was not able to distinguish patients with active TB from those without TB among symptomatic individuals undergoing investigation for TB. Two fundamental problems contributed to this outcome: a high background dermal reaction to the placebo patch and the lack of a consistent differential MPT64 reaction among TB patients.

The main analysis was conducted using efficacy rather than effectiveness because of a concern about reactions to placebo patches recorded in a pilot trial (data not shown). Measuring efficacy removed cases where a positive reaction may have been the result of irritation or reaction to the buffer. Despite this advantage, the test performed indistinguishably from random guessing. Results were unchanged by TB definition, reader training or after adjustment for age, sex and HIV status.

These results differ significantly from smaller trials conducted elsewhere,^15^,^16^ despite similar procedures and patch reagents. Although the MPT64 dose delivered here was higher than in previous trials, data from 2003 demonstrated that higher doses increased the reaction, so this was unlikely to have caused a null result.^15^ Furthermore, dosing differences would not explain the high number of positive placebo patches.

The biggest possible source of discrepancy was the difference in protein source. rMPT64 is identical to MPB64 at the amino-acid level, and has also been shown to induce an identical delayed-type hypersensitivity reaction in guinea pigs.^5^ This makes differences in reaction due to the protein unlikely. However, further analyses of the differences between MPT64 and rMPT64 have been conducted by Sequella since we completed this study. It was determined that the quaternary structure of the protein was preserved and that there were no detected post-translational modifications in either sample, although methylation was not investigated. However, two other molecules, one very small, the other 14 kDa, were found in the original sample of MPT64 tested by Nakamura et al. This sample also demonstrated significant limulus amoebocyte lystate activity, which could mean it had endotoxin-like activity or that it was contaminated with lipopolysaccharide at some point during or after purification. A novel batch of MPT64 prepared in the same manner as the original had neither the endotoxin-like activity nor the two smaller molecules; this novel preparation of MPT64 has not yet been tested clinically, but the results could point to novel patch test reagent targets.

An explanation is also needed for the frequent positive reactions to placebo patches, which significantly undermined any likelihood that MPT64 patches would helpfully differentiate TB from non-TB patients. All previous studies were conducted in the Philippines, and the significant ethnic differences might underlie different immunological responses, although, if this is found to be the case, such a phenomenon would have major implications for the global development of an immunodiagnostic of this type.

The present study had important limitations that affected the generalisability of the results. These limitations were primarily related to the population sampled. Almost everyone in the study was *mestizo*, which potentially limited the results to that specific subpopulation, even within Peru. However, far more participants were recruited into the present study than all previous studies of MPT64 patch tests combined, and the efficacy of the patch test was able to be thoroughly examined using randomised allocation, blinded reading and parallel placebo patches. Various alternative readers and reading scores were explored and patch test findings were consistently disappointing, with important implications for future research into MPT64 diagnostic tools.

## Footnotes

^*^ The appendix is available in the online version of this article, at http://www.ingentaconnect.com/content/iuatld/ijtld/2018/00000022/00000006/art00009
